# Supplementary figures and images for: T3SS effector VopL inhibits the host ROS response, promoting the intracellular survival of Vibrio parahaemolyticus
Source: PLoS Pathog. 2017 Jun 22;13(6):e1006438. doi: 10.1371/journal.ppat.1006438 (PMC5481031; doi:10.1371/journal.ppat.1006438)

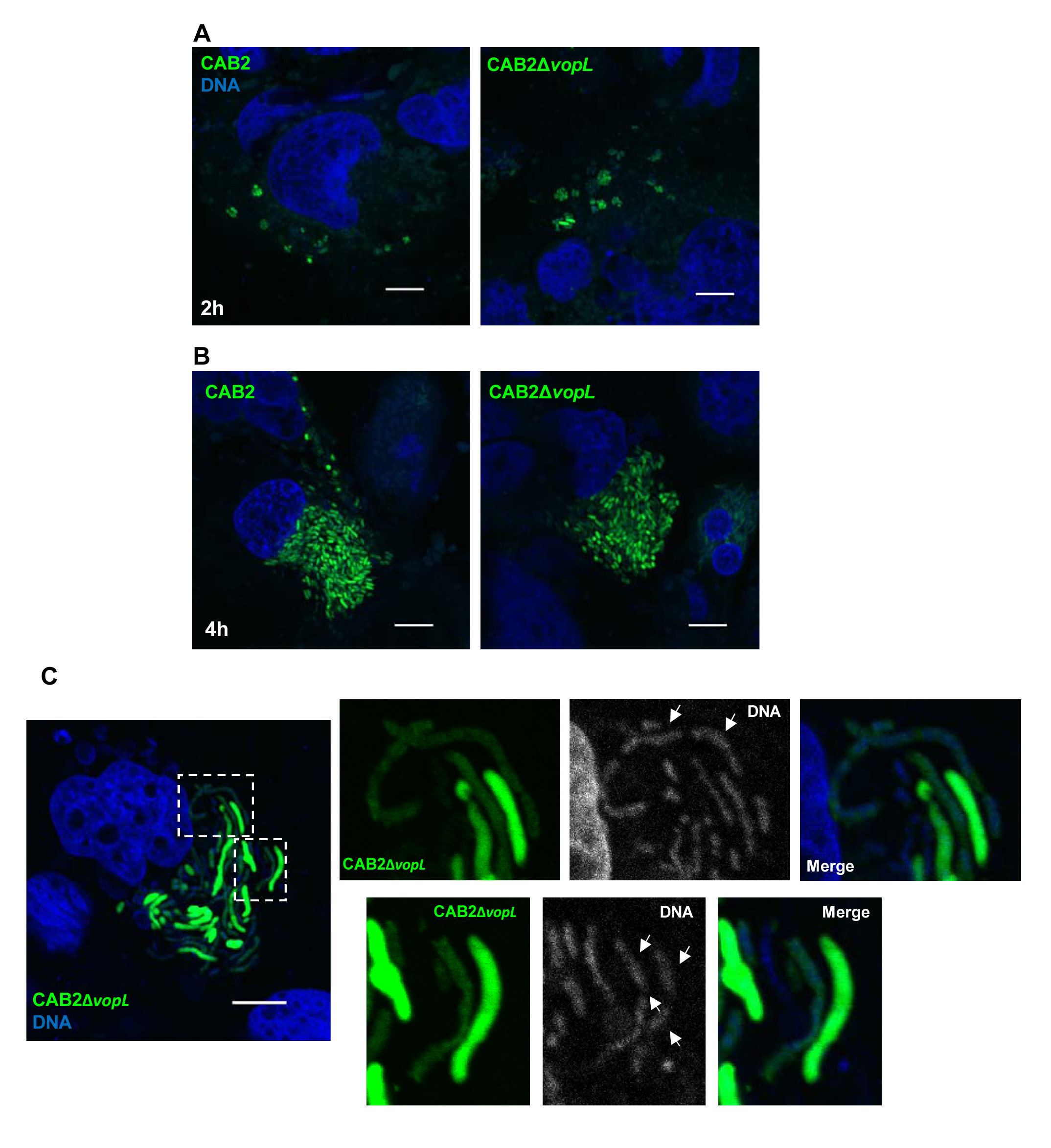

Supplement: S1 Fig — (A,B) Confocal micrographs of Caco-2 cells infected with indicated GFP-tagged CAB2 strains for 2h and incubated with 100 μg/mL gentamicin for (A) 2h or (B) 4h. DNA was stained with Hoechst (blue). Scale bars, 10 μm. (C) Confocal micrograph highlights filamentous bacteria containing multiple nucleoids. Dotted white boxes indicate magnified bacteria. White arrows indicate bacterial nucleoids. (TIF) [file ppat.1006438.s001.tif]

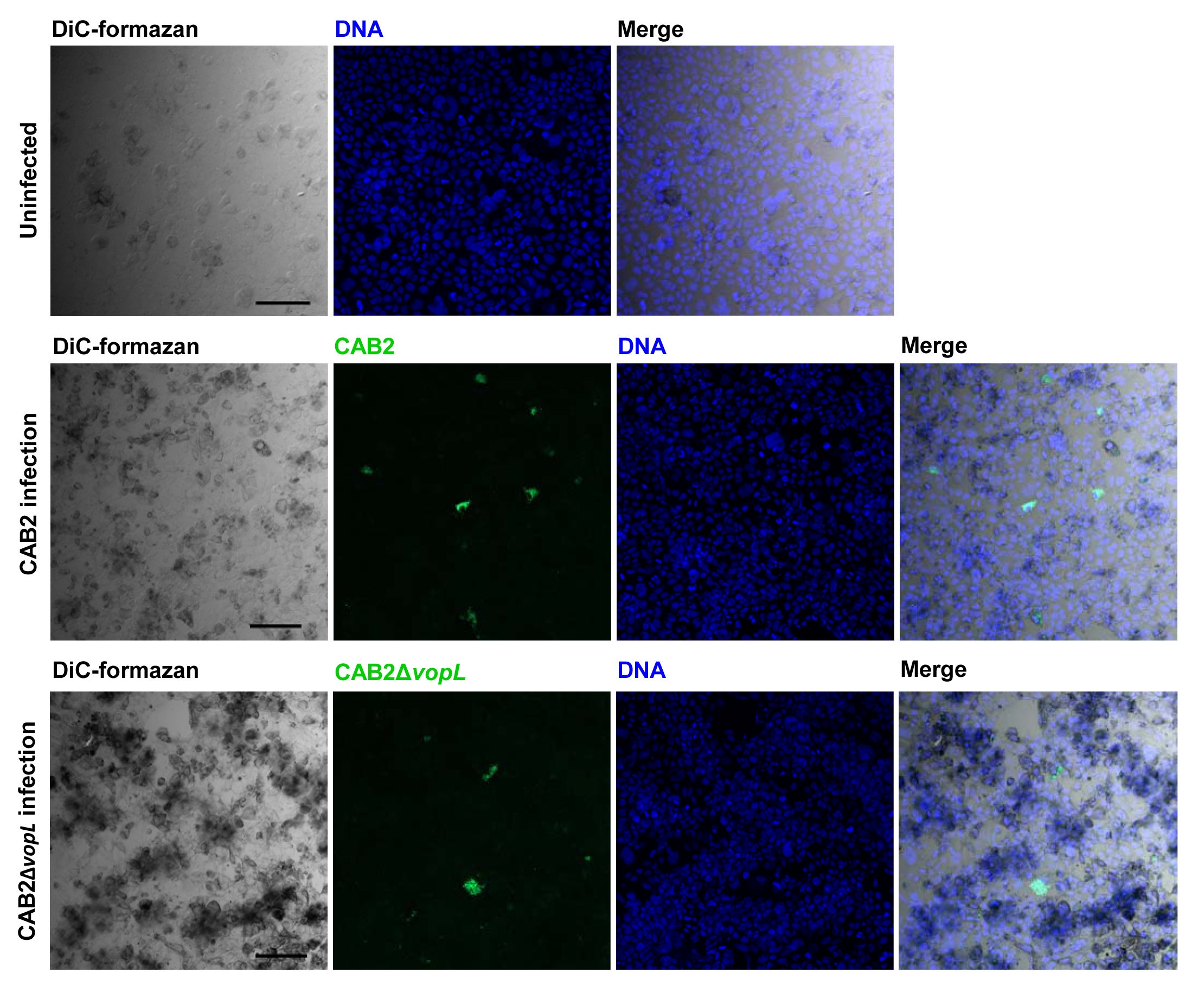

Supplement: S2 Fig — Confocal micrographs of Caco-2 cells left uninfected or infected with either CAB2-GFP (green) or CAB2ΔvopL-GFP (green) for 2h followed by incubation with 100 μg/mL gentamicin for 3h. Samples were then incubated with 1 mg/mL NBT for additional 3h in the presence of gentamicin. DNA was stained with Hoechst (blue). Formazan precipitates were visualized in bright field (DiC). Scale bars, 100 μm. (TIF) [file ppat.1006438.s002.tif]

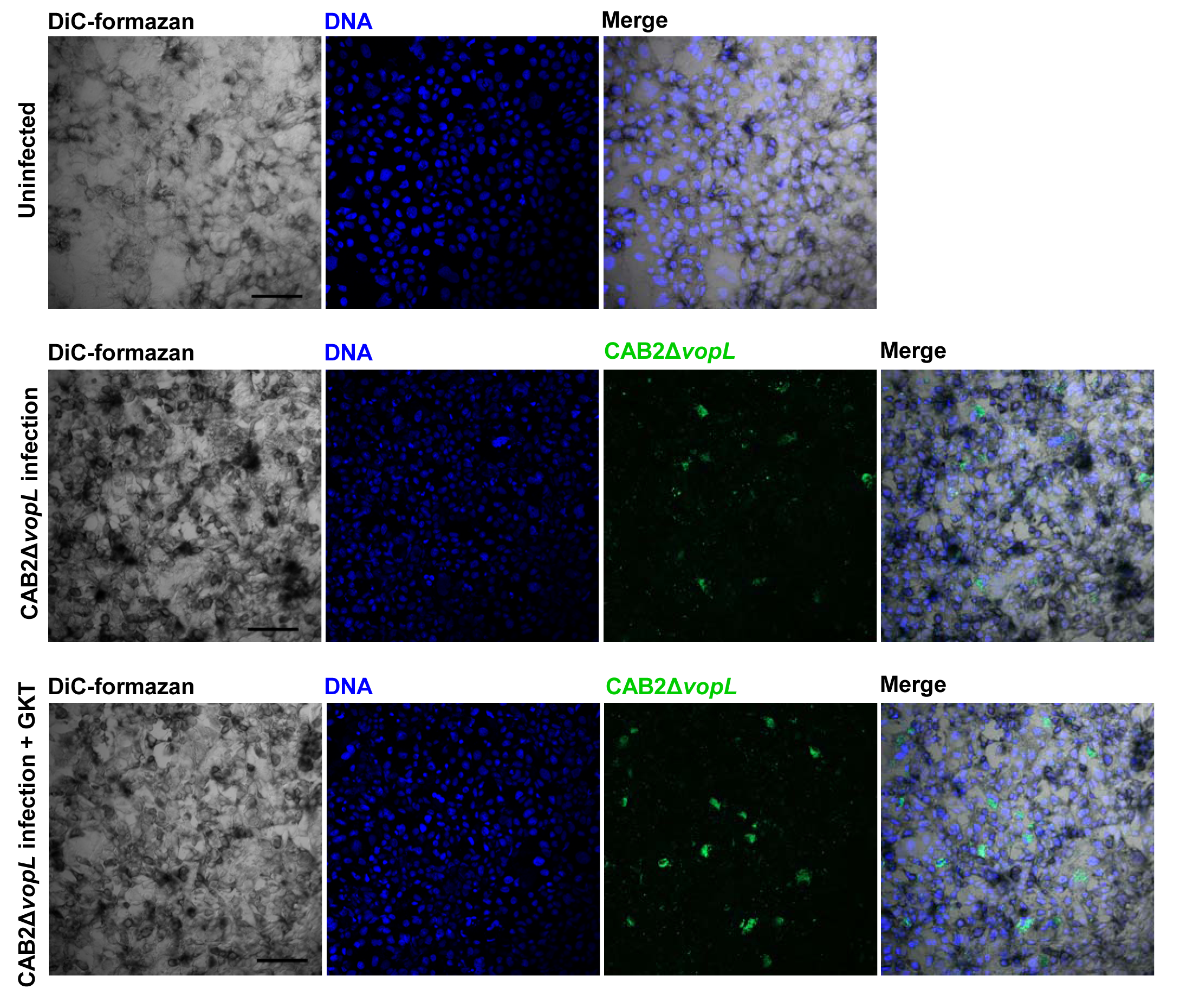

Supplement: S3 Fig — Confocal micrographs of Caco-2 cells left uninfected or infected with CAB2ΔvopL-GFP (green) for 2h followed by incubation with 100 μg/mL gentamicin for 3h. Samples were then incubated with 1 mg/mL NBT for additional 3h in the presence of gentamicin. Host cells were pre-treated with either dimethyl sulfoxide (DMSO) or 10 μM GKT136901 (GKT), which were kept throughout infection. DNA was stained with Hoechst (blue). Formazan precipitates were visualized in bright field (DiC). Scale bars, 100 μm. (TIF) [file ppat.1006438.s003.tif]

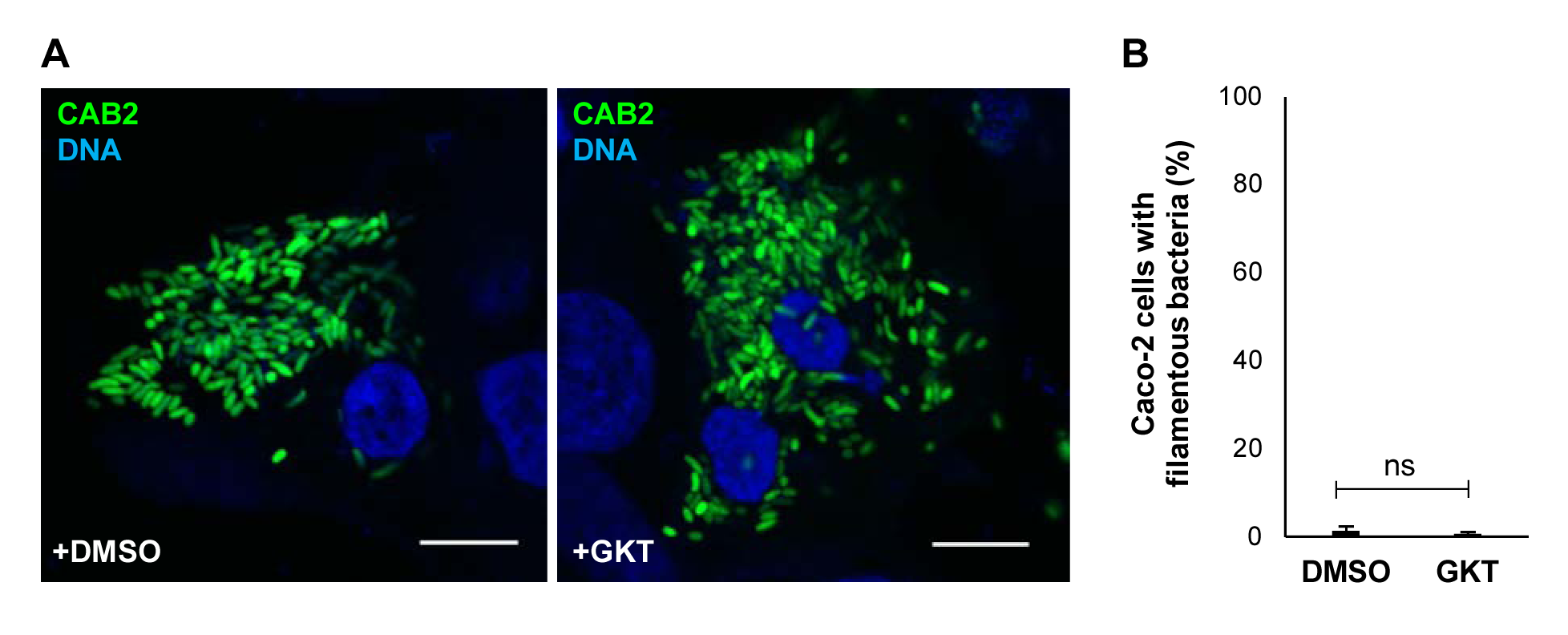

Supplement: S4 Fig — (A) Confocal micrographs of Caco-2 cells infected with CAB2-GFP (green) for 2h followed by incubation with 100 μg/mL gentamicin for 6h. Host cells were pre-treated with either dimethyl sulfoxide (DMSO) or 10 μM GKT136901 (GKT), which were kept throughout infection. DNA was stained with Hoechst (blue). Scale bars, 10 μm. (B) Quantification of filamentous bacteria in the presence or absence of GKT. Caco-2 cells invaded by CAB2-GFP and treated with either DMSO or GKT were analyzed for presence of filamentous bacteria. 300 cells for each sample (DMSO or GKT), over 3 independent experiments, were analyzed for presence of filamentous bacteria. Values are means ± SD. Difference in number of Caco-2 cells containing filamentous bacteria between DMSO- and GKT-treated samples was not statistically significant. (TIF) [file ppat.1006438.s004.tif]

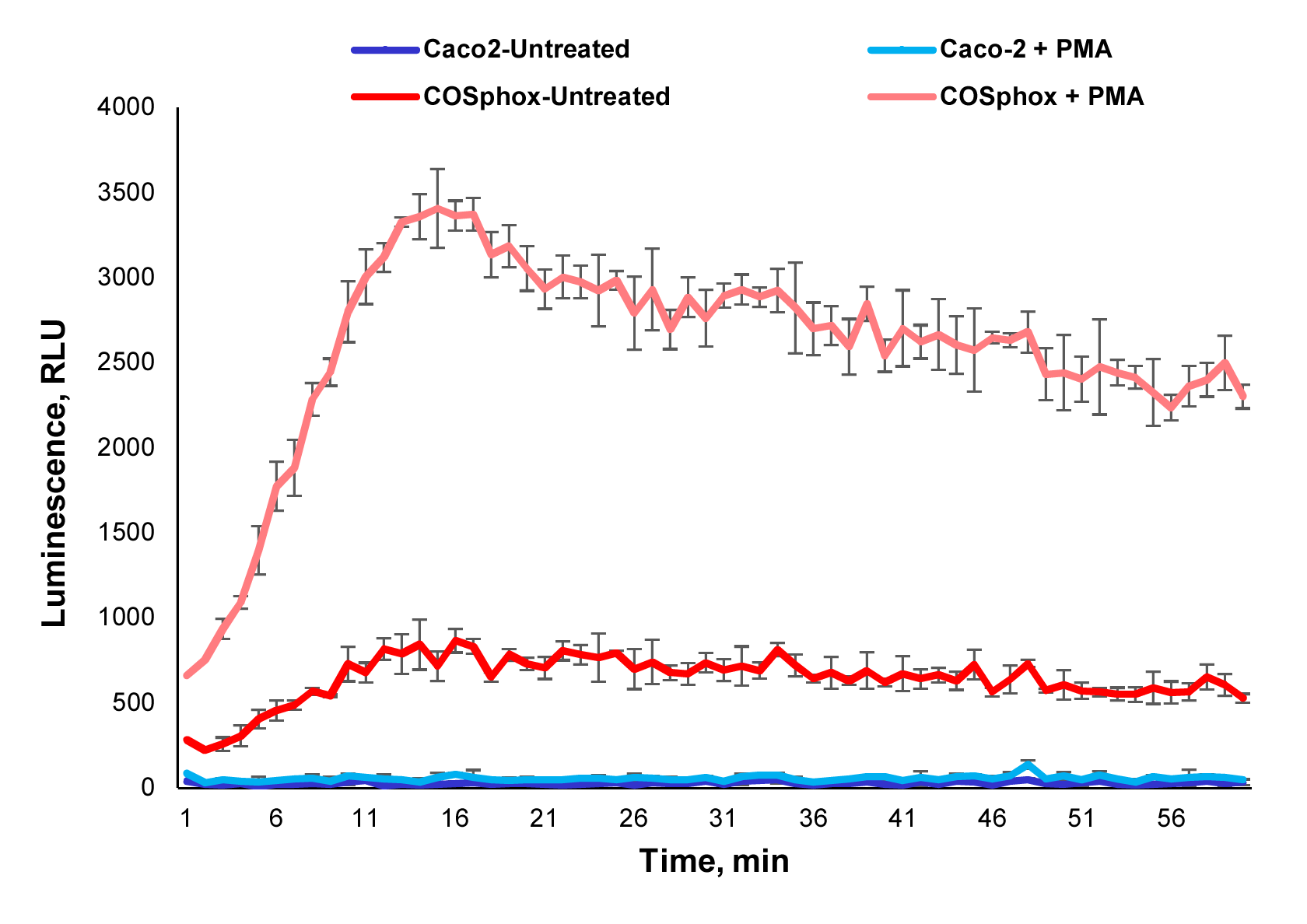

Supplement: S5 Fig — Caco-2 and COSphox cells were left untreated or treated with 0.4 μg/mL phorbol 12-myristate 13-acetate (PMA) and superoxide production was measured as a function of luminescence intensity. Values are means ± SD from one representative experiment. (TIF) [file ppat.1006438.s005.tif]

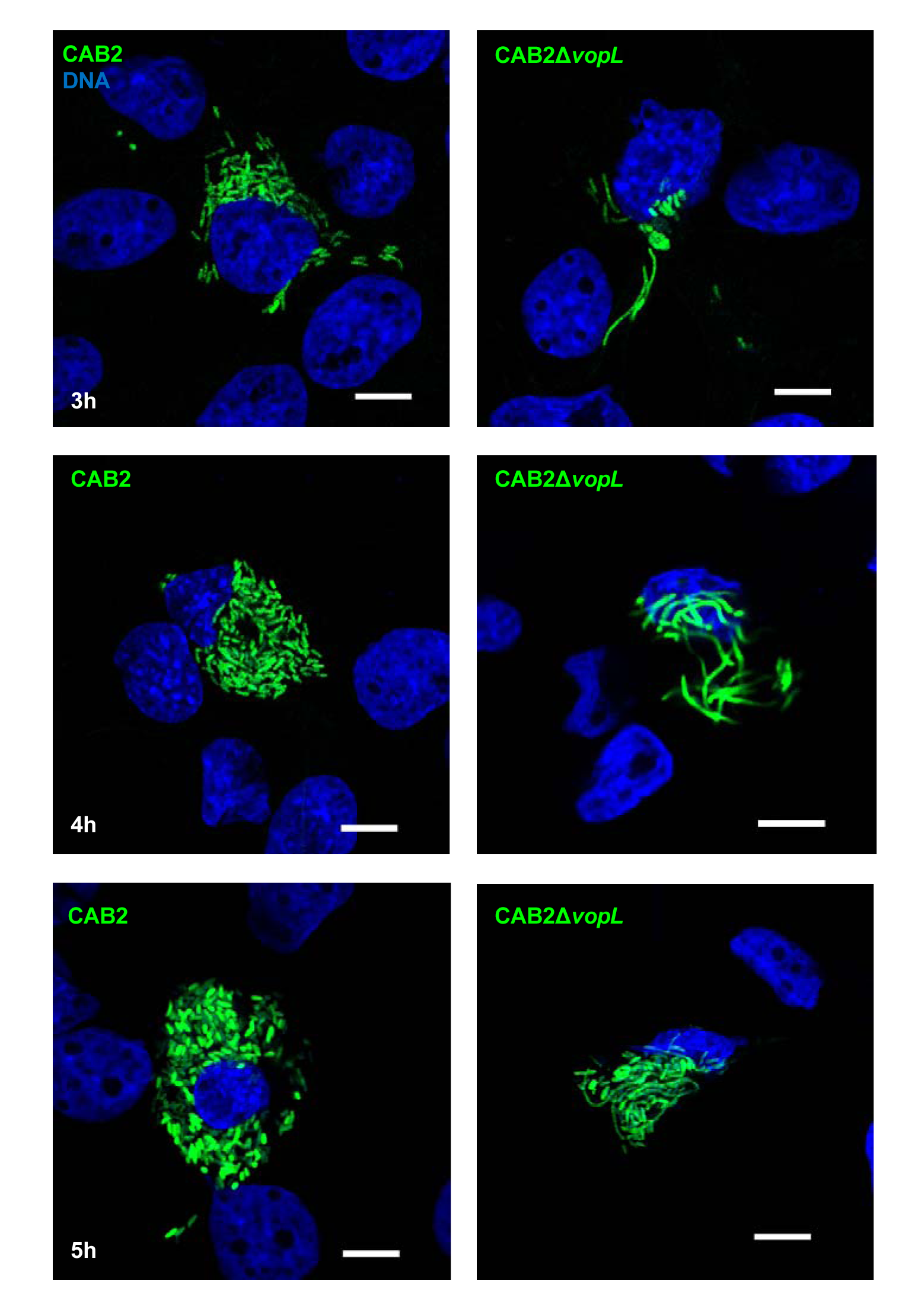

Supplement: S6 Fig — Confocal micrographs of Caco-2 cells infected with indicated GFP-tagged CAB2 strains for 2h and incubated with 100 μg/mL gentamicin for 3-5h. DNA was stained with Hoechst (blue). Scale bars, 10 μm. (TIF) [file ppat.1006438.s006.tif]

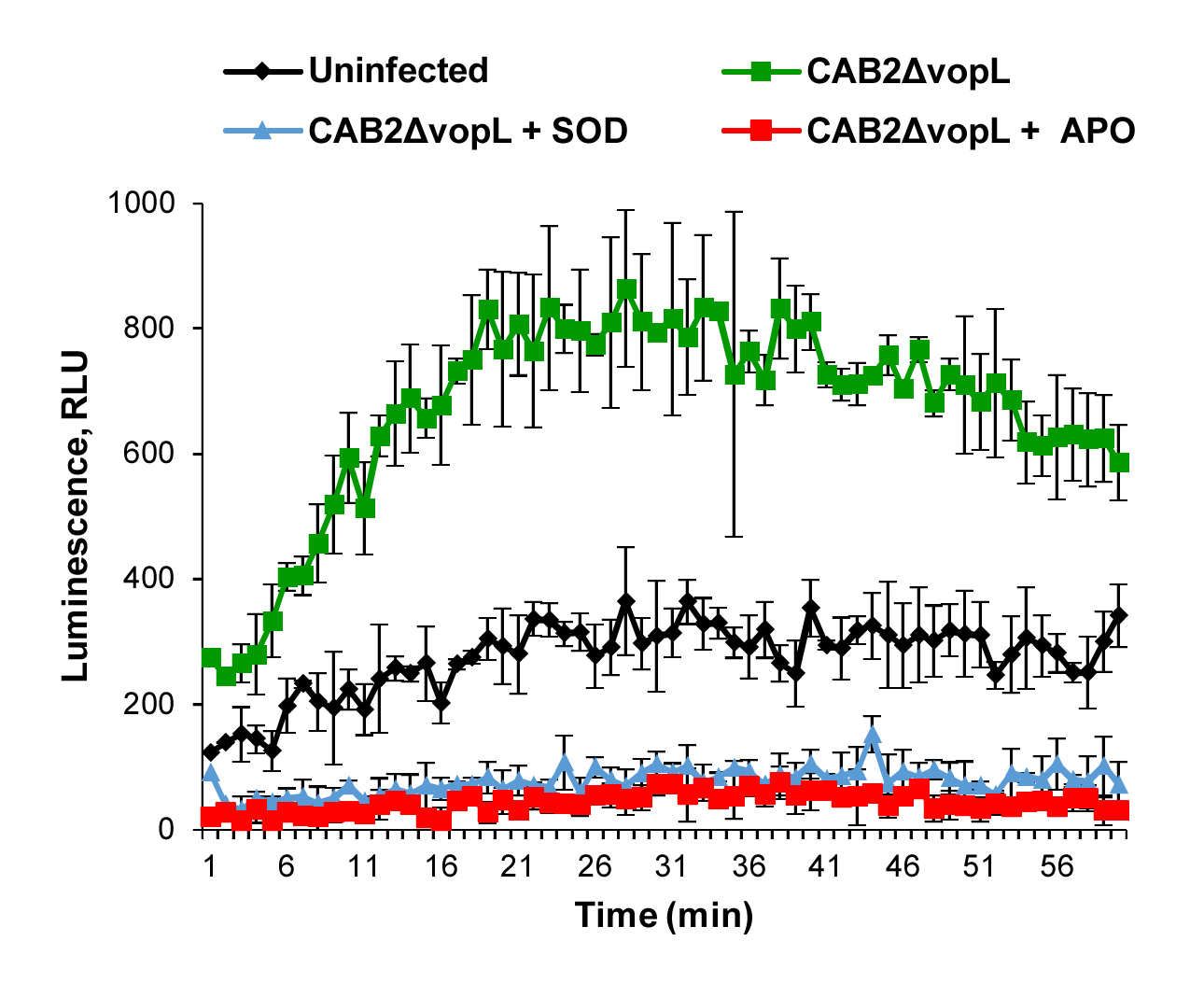

Supplement: S7 Fig — COSphox cells were infected with indicated CAB2ΔvopL for 2h. 1h prior to the end of the infection, 250 μM apocynin (APO) was added and superoxide production was measured as a function of luminescence intensity. As a positive control of suppression of superoxide, 50 units of superoxide dismutase were added at the end of infection. Values are means ± SD from one representative experiment. (TIF) [file ppat.1006438.s007.tif]

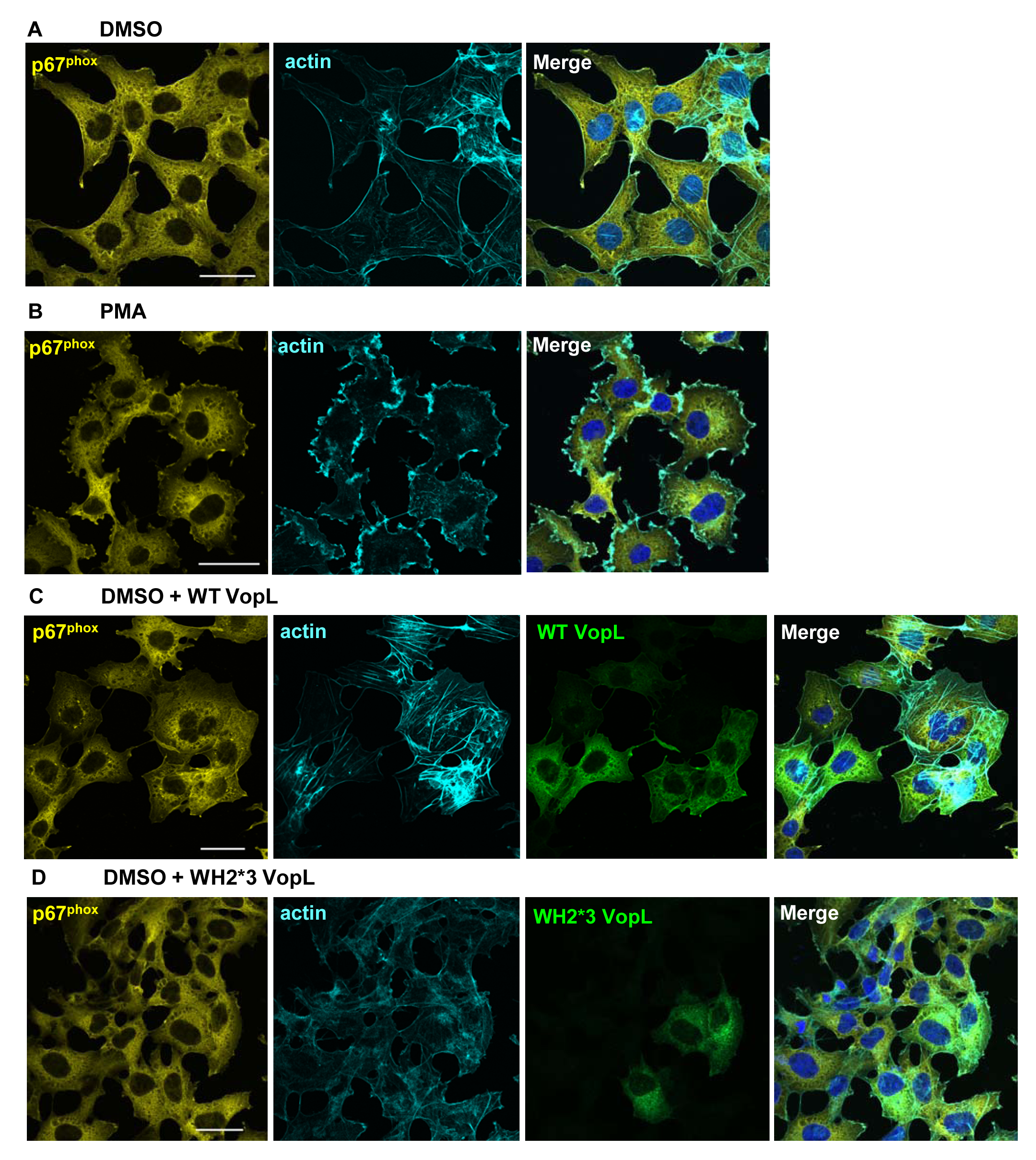

Supplement: S8 Fig — COSphox cells were stimulated for ROS production with 0.4 μg/mL phorbol 12-myristate 13-acetate (PMA, panel B). Cells treated with only vehicle (dimethyl sulfoxide, DMSO) were left unstransfected (A) or transiently transfected with either wild type VopL (WT VopL, panel C) or catalytically inactive VopL (VopL-WH2x3*, panel D). Cells were immunostained for p67phox (pseudo-colored in yellow to enhance contrast) and VopL (green). DNA and actin were stained with Hoechst (blue) and Alexa Fluor 680 phalloidin (pseudo-colored in cyan to enhance contrast), respectively. Scale bars, 40 μm. (TIF) [file ppat.1006438.s008.tif]

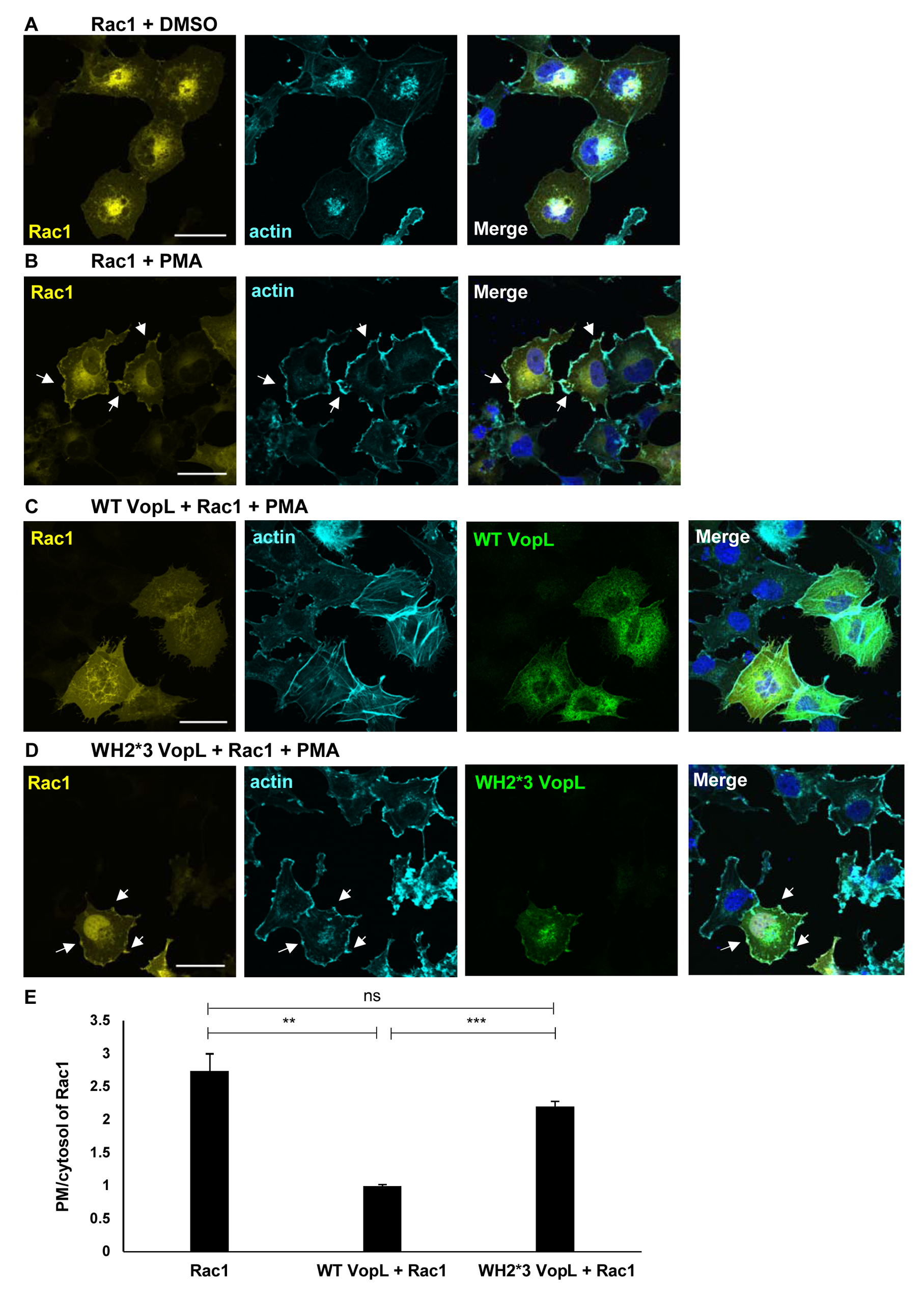

Supplement: S9 Fig — COSphox cells were transiently transfected with EGFP-Rac1 and treated only with vehicle (DMSO, A) or stimulated with 0.4 μg/mL phorbol 12-myristate 13-acetate (PMA, B). Additionally, PMA-stimulated cells were transiently transfected with either wild type VopL (WT VopL, panel C) or catalytically inactive VopL (WH2*3-VopL, panel D). Cells were immunostained for VopL (pseudo-colored in green to enhance contrast). EGFP-Rac1 was pseudo-colored in yellow to enhance contrast. DNA and actin were stained with Hoechst (blue) and Alexa Fluor 680 phalloidin (pseudo-colored in cyan to enhance contrast), respectively. Scale bars, 40 μm. (E) PMA-stimulated translocation of Rac1 from the cytosol to the plasma membrane in cells transfected only with Rac1 or transfected with both Rac1 and VopL WT/WH2*3 was monitored. Quantification was performed by analysis of line scans crossing the two cellular compartments. 90 cells for each population (Rac1 only or Rac1 + VopL WT/WH2*3) were analyzed over 3 independent experiments. Values are means ± SD. Asterisk indicates statistically significant difference between Rac1 and Rac1 + VopL WT transfected cells (** p = 0.0074) as well as between Rac1 and Rac1 + VopL WH2*3 transfected cells (*** p = 0.0005). (TIF) [file ppat.1006438.s009.tif]
